# Trace Element Uptake by Herbaceous Plants from the Soils at a Multiple Trace Element-Contaminated Site

**DOI:** 10.3390/toxics7010003

**Published:** 2019-01-17

**Authors:** Obinna Elijah Nworie, Junhao Qin, Chuxia Lin

**Affiliations:** 1 School of Environment and Life Science, University of Salford, Greater Manchester M5 4WT, UK; O.Nworie@edu.salford.ac.uk (O.E.N.); qinjunhao2015@gmail.com (J.Q.); 2 Key Laboratory of Agro-Environment in the Tropics, Ministry of Agriculture, South China Agricultural University, Guangzhou 510642, China

**Keywords:** plant uptake, trace element, contaminated site, environmental risk, soil

## Abstract

The uptake of trace elements by wild herbaceous plants in a multiple trace element-contaminated site was investigated. The bioaccumulation factor (BF) of trace elements was markedly variable among the different plant species. On average, the BF for various trace elements was in the following decreasing order: Zn > Cu > Mn > Ni > As > Pb > Cr. The translocation factor among the investigated plant species was also considerably variable and showed the following decreasing order: Mn > Zn > Ni > Cu > Cr > As > Pb. Several hyperaccumulating plants were identified: *Artemisia vulgaris* for As, Mn and Zn, *Phalaris arundinacea* for Mn and Ni, *Heracleum sphondylium* for Cr and Zn, and *Bistorta officinalis* for Mn and Zn. The marked accumulation of trace elements in the plant tissue suggests that the site may not be suitable for urban agricultural production. The plant tissue-borne trace elements could affect microbial activities and consequently interfere with the ecosystem functioning in the affected areas.

## 1. Introduction

Soil-plant translocation of trace elements is a key step leading to entry of these potentially toxic substances into the food chain [1]. Uptake of toxic elements by plants growing in contaminated soils represents a potential risk for both humans and animals due to accumulation of these trace elements in food chain [2,3]. In contaminated lands, excessive amounts of trace elements may be taken and accumulated in the above-ground portion of plants, which could lead to entry of trace elements into the food chain [4]. Trace elements which are available for plant uptake are those that exist mostly in soluble forms in soil solutions [5]. Empirical models that are effective for field applications, and that take into account plant characteristics and soil factors, have been successfully used to predict concentrations of heavy metals in plants [6]. A study by Adams et al. [7] predicted the concentrations of Cd in wheat grain based on the relationship between Cd concentration in wheat grain and soil pH. Similarly, Cao et al. [8] successfully predicted heavy metal concentrations in rice grain using a combination of soil pH and EDTA-extractable Cd, Zn, Cr, Pb and Cu concentrations in the soils. Warne et al. [9] also found that the use of soil properties to estimate phytotoxic effects of heavy metals across different soil-crop systems was one of the fundamental steps in improving risk assessment of heavy metals in soil.

Plant uptake of trace elements could pose a risk to wild animals, grazing cattle and humans who are exposed to the contaminated plants [10]. To assess the environmental risk from the exposure to contaminated plant tissues in a contaminated site, it is necessary to understand the status of trace elements in soil-plant systems. Although there has been some work on trace element uptake by plants in contaminated soils, previous studies have focused on the phytoextracting capacity of hyperaccumulating plants in greenhouse settings. There have been only a few studies being conducted under field conditions. For example, Nguyen et al. [11] reported that Eleocharis acicularis hyper-accumulated a significant amount of Pb in an abandoned mine site. Similarly, Ranjeev et al. [12] found that Eichhornnia crassipes accumulated considerable amounts of Fe, Mn and Cu in a site contaminated with industrial effluents. Furthermore, Yoon et al. [4] found that various herbaceous plants investigated in a contaminated site in Florida had the capacity to accumulate Pb, Cu and Zn.

Although there has been some work done on trace element uptake by plants in contaminated soils, there is still a paucity of information on the uptake and translocation of trace elements by herbaceous plants in multi-contaminated lands. This study aims to evaluate the uptake of various trace elements by a range of herbaceous plants and the associated environmental risk in a historical landfill site with industrial wastes being disposed of during the Industrial Revolution. It was intended to be a preliminary baseline study to provide information for selection of the long-term monitoring sites and conduct of greenhouse experiments to explore the insights into the mechanisms responsible for the plant uptake of the investigated trace elements.

## 2. Materials and Methods

### 2.1. The Study Site

**Figure 1:**
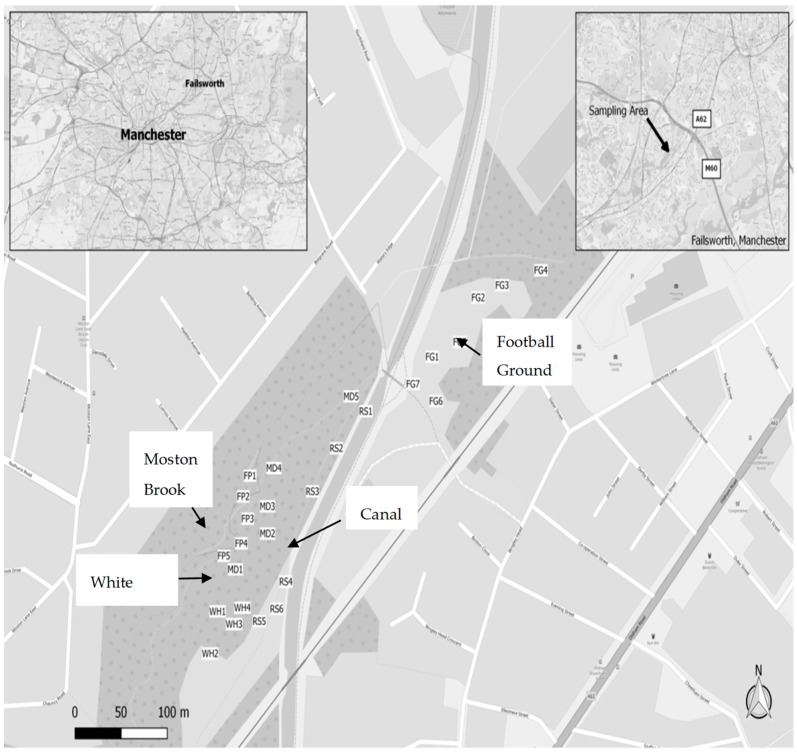
Map showing the study area and sampling locations.

The study site is part of the Moston Brook area that is situated in the Greater Manchester region, England (latitude: 53.515889° N; longitude: 002.155625° W) (Figure 1).

During the period of Industrial Revolution, the Moston Brook area was part of the “cottonopolis” with dyeing and bleaching of finished cotton fabrics, and printing and brick making being the major industrial activities in the area [13]. The investigated area is part of a closed landfill site that received various solid industrial wastes in the past. Since the disposal of wastes took place prior to the enactment of the Pollution Control Act in 1974, it is practically impossible to track down the history of landfill operations. However, an area of contamination (locally known as “White Hills”) has been identified between the canal and the brook with the soils consisting of pale-yellow materials. Contamination is suspected to extend in a north-south direction and there is a concern that runoff and erosion from the exposed bank is impacting the water and sediment quality of the brook. Another area of contamination (locally known as “Football Ground”) that is largely unvegetated has also been identified, which is located on the opposite side of the canal from the “White Hills” [13]. The site is currently being used as a public open space for the local people and visitors and the local authorities have proposed to re-develop the site for recreation and other beneficial uses [13].

### 2.2. Field and Laboratory Methods

Twenty-seven herbaceous plants (representing 26 dominant species) with their corresponding soils were collected across the study area in May 2015. The sampling locations are shown in Figure 1. Samples WH1-4 were collected from the central part of the “White Hills”; Samples MD1-5 were collected from the area extending north from the central part of the “White Hills”; Samples FP1-5 were collected from the floodplain of the Moston Brook; Samples RS1-6 were collected from the soils along the road running parallel to the canal; and Samples FG1-7 were collected from the “Football Ground”. At each location, the plant with its corresponding rhizospheric soil was taken using a spade. The samples were then put in appropriately labelled polyethylene bags and immediately transported to the laboratory on the same day for pretreatment.

In the laboratory, the plants were carefully separated from the soils. The soil samples were then oven-dried at 40 °C, ground to pass a 2 mm sieve and stored in sealable laboratory bags prior to analysis. The plant samples were washed with deionized water to remove all the soils attached to the roots, dried with a paper towel and air-dried for about two hours. The air-dried plant samples were then separated into roots and shoots and the fresh biomass of both root and shoot portions were measured. The fresh plant parts were contained in paper bags and oven-dried at 65 °C for three days. After oven-drying, the dry biomass of root and shoot portions was determined. Each of the dried plant samples was then ground using an electrical plant tissue pulverizer and stored in a sealable bag prior to analysis.

Soil pH and electrical conductivity (EC) were measured in 1:5, soil:water extracts using a calibrated pH meter and EC meter, respectively. The concentration of trace elements contained in the soils was determined by X-ray fluorescence microscopy (Niton XL2 Gold Handheld XRF Analyser, Winchester, UK). The analytical procedure is as follows: a sub-sample was further ground to pass a 0.063 mm sieve; the samples were agitated for 5 min by mechanical sieve shaker and then 5 g of each of the soil samples was transferred into an XRF sampling cup; a thin cling film sheet was used to cover the base of the cup prior to XRF analysis. During XRF analysis, the XRF analyser was placed on holding support and then connected to a laptop through which real time data of the analysis was obtained in an excel file. The instrument was calibrated by analysing the 73,308 standard reference materials prior to proper analysis. To ensure accuracy and reliability of the results obtained, all analyses were performed in duplicates and the analysis time was 240 s.

The concentration of trace elements in the plant tissues was determined by ICP-OES (Varian 720ES ICP-OES, Palo Alto, CA, USA). For each plant tissue sample, 0.5 g of the powdered sample was weighed and added into a microwave digestion tube, followed by adding 1 mL of 30% (*m*/*m*) H_2_O_2_ and 7 mL of concentrated HNO_3_ solution. The tubes were fitted with bungers and closed appropriately with lids to prevent loss of volatile elements during digestion and placed into microwave carousel. The microwave digestion was done by firstly increasing the temperature linearly from 25 to 90 °C for 4 min; secondly, the temperature was maintained steadily at 90 °C for 2 min; thirdly, the temperature was increased linearly to 180 °C for over 6 min; and finally, the temperature was maintained at 180 °C for 10 min [14]. After cooling, the digested plant samples were filtered using Whatman filter papers (No. 42). The filtrates were then diluted to a final volume of 25 mL with deionized water. The diluted samples were stored at 4 °C in a refrigerator prior to analysis of trace elements using ICP-OES.

## 3. Results

### 3.1. pH and EC

The pH and EC of the investigated soils are presented in Table 1. pH ranged from 5.46 to 8.32 with a median value of 6.50. The median of EC was 64.5 µS/cm with a range of 17.6–182. No clear spatial variation trend is observed though the pH, and EC tended to be lower in the WH portion (WH1-4) and tended to be higher in the FP portion, as compared to other portions. There is no close relationship (*R*^2^ = 0.26, *n* = 27) between the pH and EC.

### 3.2. Total Concentration of Trace Elements in the Investigated Soils

Various trace elements in the investigated soils were spatially variable. In terms of median concentration, they were in the following decreasing order: Mn > Pb > As > Cr > Zn > Cu > Ni. There were a few spots with concurrent presence of Cu, Mn, Ni, Pb and Zn at very high concentration (FG1, FG2, FG5 and RS3). Most of the soils had a concentration of Cr over 100 mg/kg. There were seven spots where the concentration of As was greater than 500 mg/kg (Table 2).

### 3.3. Concentration of Trace Elements in Plant Tissues

#### 3.3.1. Roots

The concentration of trace elements in the roots of the investigated plant species is given in Table 3. For the investigated plant species, the concentration of trace elements in the root portion varied markedly from plant species to plant species. The median concentration of root-borne trace elements was in the following decreasing order: Mn > Zn > Cu > Pb > Ni > As > Cr.

Root-borne As had a median concentration of 5 mg/kg with a range of 1–103 mg/kg. Among the investigated plant species, *Bistorta officinalis* and *Lolium pratense* had the first and second highest concentration of As in the root portion. *Dactylis glomerata* and *Urtica dioica* also had relatively higher concentration of root-borne As, as compared to most of the investigated plant species. In contrast, some plant species such as *Artemisia vulgaris*, *Chamerion angustifolium* and *Plantago lanceolata* showed very low concentration of root-borne As (<2 mg/kg). The root-borne Cr ranged from under detection limit to 46 mg/kg with a median concentration of 3 mg/kg. *Dactylis glomerata* had the highest concentration of Cr in the root portion while *Galium aparine* had no detectable Cr in the plant roots. *Heracleum sphondylium*, *Impatiens glandulifera*, *Holcus lanatus* also showed very low root-borne Cr concentration (<1 mg/kg). The root-borne Cu ranged from 9 mg/kg to 731 mg/kg with a median concentration of 42 mg/kg. *Agrostis tenuis* had the highest concentration (731 mg/kg) of root-borne Cu while *Heracleum sphondylium* had the lowest concentration of Cu (9 mg/kg). The concentration of root-borne Mn ranged from 25 to 584 mg/kg with a median concentration of 182 mg/kg. *Phleum pratense* had the highest concentration of root-borne Mn among the investigated plant species. The lowest concentration of root-borne Mn was observed in *Heracleum sphondylium* with a concentration of 25 mg/kg. The root-borne Ni ranged from 1 to 317 mg/kg among all the investigated plant species. The median root-borne Ni was 33 mg/kg. The highest root-borne Ni concentration (317 m/kg) was found in *Agrostis tenuis*, followed by *Agrostis stolonifera*. The lowest root-borne Ni concentration was recorded in *Galium aparine*. The concentration of root-borne Pb ranged from 3 to 256 mg/kg among the investigated plant species. The median concentration of root-borne Pb was 23 mg/kg. *Agrostis tenuis* had the highest concentration of root-borne Pb, followed by *Agrostis stolonifera*. The lowest concentration of root-borne Pb was observed in *Chamerion angustifolium*. The root-borne Zn had a median concentration of 122 mg/kg with a range of 50–558 mg/kg. *Agrostis tenuis* had the highest concentration of root-borne Zn while the lowest concentration was held by *Chamerion angustifolium*.

#### 3.3.2. Shoots

The concentration of trace elements in the shoots of the investigated plant species is shown in Table 4. Like roots, it varied from plant species to plant species. The median concentration of various shoot-borne trace elements among all the investigated plant species are in the following decreasing order: Zn > Mn > Cu > Ni > Pb > As > Cr, which is similar to that in the root portion. For As, the shoot-borne concentration ranged from UDL to 6.2 mg/kg with a median concentration of 0.7 mg/kg. *Phleum pratense* had the highest shoot-borne As while the lowest shoot-borne As (UDL) was found in *Dactylis glomerata*. Other investigated plant species (*Agrostis stolonifera*, *Argrostis capillaries*, *Chamerion angustifolium*, *Urtica dioica*, *Galium aparine*, *Phalaris arundinacea*, *Heracleum sphondylium*, *Impatiens glandulifera*, *Plantago lanceolate*, *Lolium multiflorum*, *Lolium pratense*, *Equisetum arvense*, *Cynosurus cristatus* and *Filipendula ulmaria*) also had a very low As concentration in their shoots (<1 mg/kg). The shoot-borne Cr ranged from UDL to 0.8 mg/kg with a median concentration of 0.4 mg/kg. *Bistorta officinalis* had the highest shoot-borne Cr (0.8 mg/kg) while *Chamerion angustifolium* contained the least amount of shoot-borne Cr. The shoot-borne Cu ranged from 3.5 to 58.7 mg/kg with a median concentration of 8.5 mg/kg. The shoot-borne Mn ranged from 9.1 to 540 mg/kg with a median concentration of 37.5 mg/kg for the investigated plant species. *Bistorta officinalis* contained the highest amount of shoot-borne Mn, followed by *Phleum pratense* while the lowest concentration of shoot-borne Mn was found in *Urtica dioica*. The shoot-borne Ni ranged from 0.8 to 52.8 mg/kg. The mean concentration of Ni was 2.0 mg/kg. *Agrostis tenuis* contained the highest amount of Ni in the shoot, followed by *Artemisia vulgaris*. The lowest shoot-borne Ni was found in *Galium aparine* that had only 0.8 mg/kg. The concentration of Pb in the shoots ranged from 0.2 to 27.8 mg/kg with a median concentration of 1.79 mg/kg. The highest concentration of shoot-borne Pb was found in *Agrostis tenuis* while *Chamerion angustifolium* had the lowest Pb concentration in the shoot among the investigated plant species. For shoot-borne Zn, the concentration ranged from 14.5 to 189 mg/kg with a median value of 39.5 mg/kg. *Artemisia vulgaris* contained the highest amount of Zn in the shoot, followed by *Agrostis tenuis*. *Holcus lanatus* had the lowest amount of Zn in the shoot among the investigated plant species.

## 4. Discussion

### 4.1. Trace Elements in the Investigated Soils

From Table 2, it can be seen that the median concentration of As, Cu, Pb and Zn was about 30-, 4.5-, 9.6- and 1.8-fold higher than that in typical UK soils, respectively while Mn and Ni had the median concentration very similar to the respective UK-wide value [15]. The median concentrations of soil-borne As and Pb all well exceed the current UK screening levels for these two regulated soil elements [16]. There were a few spots with concurrent presence of Cu, Mn, Ni, Pb and Zn at very high concentration (FG1, FG2, FG5 and RS3). Most of the soils had a concentration of Cr over 100 mg/kg. There were seven spots where the concentration of As was greater than 500 mg/kg (Table 2). Pb concentration in the investigated soils were within the range previously reported by others [15,17,18,19] for British soils except in FG1, FG2, RS3 and WH1 soils where the observed Pb concentration was above the reported concentration range, indicating elevated level of Pb in these soils. The concentration of As in the investigated soils exceeded the upper limit of background As level (74.4 mg/kg) in British soils [17] except for the soils collected at FG1, FG4, FG5, FG6, FG7, FP4, FP5 and MD1 where the soil-borne As concentration was within the background As range. Cr levels observed in the investigated soils are within the range reported in literature for UK soils. Ni concentration in most of the investigated soils fell within the range reported for the British soils [15] and the world soils (0.2–450 mg/kg) [20] except for the soils collected from Locations FG1, FG2, FG5 and RS3 where elevated Ni level was present. Cu level in the investigated soils varied widely and in most investigated soils, Cu concentration was within the range reported for British soils [15,17,21] except in FG1, FG2, FG5, FG6, FG7 and RS3 where elevated Cu levels were observed. The results indicated that Cu was among the most dominating heavy metals in FG soils. Zn concentration in most of the soils is in agreement with the range of soil-borne Zn reported for UK soils [15,18] except in FG1, FG2, FG5, FG7, FP2 and RS3 soils where elevated levels of Zn were observed. However, McGrath et al. [22] did report extremely high Zn concentration (3648 mg/kg) for some UK soils.

There was no clear relationship (*R*^2^ < 0.1) between the concentration of root-borne trace elements and pH, suggesting that uptake of trace elements by plant roots was not controlled by soil pH. Possibly, the low-molecular-weight organic acids released from the roots played a more important role in mobilizing the soil-borne trace elements in rhizosphere and thus making the trace elements available for the uptake by the plant roots.

### 4.2. Bioaccumulation of Trace Elements

Bioaccumulation factor, according to Zhao et al. [23], is defined as the capacity of a plant to accumulate a specific trace element with relation to its concentration in the soil. The ability of plants to take trace elements from soils can be quantified using bioaccumulation factor (BF). Bioaccumulation factor of trace elements in this study was calculated as a ratio of trace element concentration in plant root to that in the soil:

BF = C_plant root_/C_soil_ (1)

where C_plant root_ and C_soil_ represent the concentration of a given trace element in plant root issue and in the corresponding soil sample, respectively.

The bioaccumulation factors of trace elements for the investigated plant species are presented in Table 5. It is evident that BF was highly variable among the different plant species growing in the soils with different physiochemical characteristics. This is consistent with work by Bose et al. [24]. The median value of BF for Zn was close to 1, indicating strong uptake of Zn by plant roots. Cu had a BF over 0.5, followed by Mn and Ni, which had a BF of more than 0.25 and nearly 0.15, respectively. In comparison, the BF for As, Cr and Pb in most of the plant species was below 0.1, indicating that these trace elements have relatively low phyto-availability, which is consistent with the findings by Tiwari et al. [25].

Some plant species showed unusual capacity to take certain trace elements from the soils. For As, *Bistorta officinalis* had much higher BF, as compared to other plant species. Three plant species had BF >1 for Cu: *Argrostis capillaries*, *Glyceria maxima*, *Filipendula ulmaria*. There were four plant species with BF >1 for Mn, including *Lolium pratense*, *Cynosurus cristatus*, *Bistorta officinalis* and *Lolium multiflorum* with the *Lolium multiflorum* having a BF >4. For Ni, *Heracleum sphondylium* and *Juncus effuses* had the BF >1. It is interesting to note that nearly half of the investigated plant species had a BF greater than 1 for Zn with *Lolium multiflorum* having a BF as high as 7.69. It has been previously reported that *Lolium multiflorum* could tolerate high concentrations of heavy metals in mine soils and has the capacity to phytoextract a range of heavy metals from soils [26]. In addition, *Dactylis glomerate* and *Bistorta officinalis* had the capacity to phytoextract a wide range of trace elements.

### 4.3. Root-Shoot Translocation

The translocation factor (TF) is used here to evaluate the translocation of trace elements from plant root to shoot [27]. The TF is calculated as a ratio of trace element concentration in plant shoot to that in plant root:

TF = C_plant shoot_/C_plant root_ (2)

The TFs for the 27 investigated plant samples are given in Table 6. In general, the TF among the investigated plant species was extremely variable. The median value of TF for various trace elements was in the following decreasing order: Mn > Zn > Ni > Cu > As > Cr > Pb. Manganese, zinc and nickel are readily transported via xylem to the shoot [28]. The high rate of root-shoot translocation for Mn Zn, Ni and Cu was also noted by other workers [29,30,31,32,33].

Several plant species had TFs near to or greater than 1 at least for one trace element and therefore can be regarded as hyperaccumulating plants [34]. These include *Artemisia vulgaris* for As, Mn and Zn, *Phalaris arundinacea* for Mn and Ni, *Heracleum sphondylium* for Cr and Zn, and *Bistorta officinalis* for Mn and Zn. It is interesting to note that *Phalaris arundinacea* has a TF as high as 5.42 for Mn, suggesting that it could be a high-performing Mn hyperaccumulator. This finding is in contrast with Polechońska and Klink [35] who found that the root-shoot translocation of heavy metals was limited.

### 4.4. Environmental Implications

**Figure 2:**
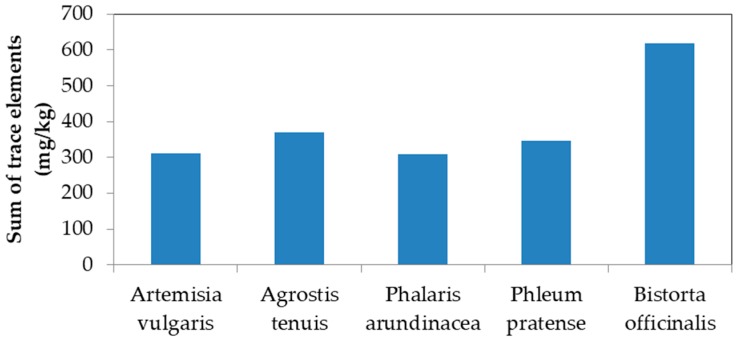
Sum of various trace elements (mmol/kg) in the shoot portion for the five top plant species.

Information on trace elements in wild plants is rare. By comparison with other available reports, it is evident that the levels of shoot-borne trace elements in the investigated study area tended to be higher than those reported by Misra and Mani [36] and MacLean et al. [37] except for As and Cr (Table 7). In particular, several plant species are capable of accumulating multiple trace elements and have a very high level of combined trace elements in the shoot portion (sum of all the investigated trace elements). These include *Artemisia vulgaris*, *Agrostis tenuis*, *Phalaris arundinacea*, *Phleum pratense* and *Bistorta officinalis* (Figure 2).

Heavy metal uptake by vegetable plants has been a topic receiving great attention due to the human health risk associated with consumption of contaminated vegetable products [38,39,40]. There has been a movement to develop urban agriculture by using available lands near residential areas such as the investigated area [41]. The marked accumulation of trace elements in some investigated plant species suggests that it is possible that certain types of vegetable plants could take substantial amounts of trace elements from the soils if the site is used as urban farms for agricultural production. Therefore, caution must be taken to evaluate the human health risk associated with any potential urban agriculture development.

Although the investigated herbaceous plants do not have direct impacts on human health due to their non-human-consumable nature, the plant tissue-borne trace elements could affect the ecosystem functioning in the affected areas. The generally high concentration of trace elements in the root could affect soil animals and microorganisms that feed on the root-derived organic matter [42,43,44,45]. If the site is used for cattle grazing, the shoot-borne trace elements could potentially affect the health of the grazing animals and accumulate in the animal tissues, which in turn affect human health through the food chain.

## 5. Conclusions

The investigated soils were contaminated by multiple trace elements with the median concentration being in the following decreasing order: Mn > Pb > As > Cr > Zn > Cu > Ni. The median concentration of trace elements in the roots of the investigated plant species was in the following decreasing order: Mn > Zn > Cu > Pb > Ni > As > Cr while the median concentration of trace elements in the shoots of the investigated plant species showed the following decreasing order: Zn > Mn > Cu > Ni > Pb > As > Cr. The bioaccumulation factor was highly variable among the different plant species. Uptake of Zn by plant roots was strong with a median BF of 0.856. Cu and Mn had a BF over 0.5 and 0.25, respectively. These were followed by Ni with a BF nearly 0.15. In comparison, As, Cr and Pb tended to have relatively low phyto-availability with the median BFs being <0.1 for most of the plant species. The translocation factor among the investigated plant species was considerably variable. In terms of median value, TF for various trace elements was in the following decreasing order: Mn > Zn > Ni > Cu > As > Cr > Pb. Potential hyperaccumulating plants identified from this study included *Artemisia vulgaris* for As, Mn and Zn, *Phalaris arundinacea* for Mn and Ni, *Heracleum sphondylium* for Cr and Zn, and *Bistorta officinalis* for Mn and Zn. The marked accumulation of trace elements in the plant tissue suggests that the site may not be suitable for urban agricultural production. The plant tissue-borne trace elements could affect the ecosystem functioning in the affected areas. The findings obtained from this study provide a basis for further work to obtain insights into plant uptake of the trace elements contained in the investigated soils through long-term field monitoring and greenhouse experiments.

## Figures and Tables

**Table 1:** pH and Electrical Conductivity of the investigated soils.

Location	pH	EC (µS/cm)
FG1	7.12	39.1
FG2	6.62	35.2
FG3	5.86	70.6
FG4	8.32	96.4
FG5	6.57	17.6
FG6	6.16	64.8
FG7	6.76	182
FP1	7.58	39.2
FP2	7.69	135
FP3	6.27	70.6
FP4	7.41	138
FP5	7.10	152
MD1	7.53	123
MD2	5.46	38.9
MD3	6.21	42.1
MD4	7.61	153
MD5	7.02	64.3
RS1	6.39	116
RS2	6.47	72.4
RS3	6.50	45.2
RS4	6.81	132
RS5	5.50	53.5
RS6	6.25	61.5
WH1	5.85	37.5
WH2	6.12	62.1
WH3	6.16	51.4
WH4	6.09	45.5
Median	6.50	64.5
Range	5.46–8.32	17.6–182

**Table 2:** Total concentration (mg element/kg dry weight) of trace elements in the investigated soils.

Location	As	Cr	Cu	Mn	Ni	Pb	Zn
FG1	69	155	2768	3855	802	1492	1263
FG2	80	154	3348	3458	920	1587	1026
FG3	770	223	59	361	UDL ^a^	649	89
FG4	12	47	197	397	55	114	186
FG5	39	121	1237	3499	1075	891	1881
FG6	40	103	408	767	145	345	218
FG7	51	133	626	1017	129	402	580
FP1	510	161	78	502	57	362	143
FP2	148	144	170	774	71	434	453
FP3	472	136	119	499	17	919	242
FP4	54	108	63	428	53	137	128
FP5	50	95	60	416	55	132	119
MD1	34	87	32	581	11	46	72
MD2	1132	126	38	49	UDL	623	28
MD3	269	142	35	340	6	191	71
MD4	761	204	79	309	UDL	690	89
MD5	215	126	57	432	11	253	121
RS1	350	165	78	351	24	337	174
RS2	451	172	52	362	22	330	95
RS3	88	177	2380	1957	425	1316	1450
RS4	324	152	75	524	9	329	118
RS5	857	202	89	2245	UDL	597	92
RS6	688	217	88	254	UDL	569	109
WH1	993	200	61	383	UDL	1380	89
WH2	96	108	134	312	10	156	95
WH3	268	139	51	466	UDL	257	107
WH4	125	117	54	394	17	148	98
Median	215	142	78	432	17	362	119
Min	12	47	32	49	UDL	46	28
Max	1132	223	3348	3855	1075	1587	1881
UK soil range ^b^	0.5–143	1.14–236	2.27–96.7	10–12,200	1.16–216	2.6–713	2.63–442
UK soil median ^b^	7.1	29.2	17.2	420	15.8	37.4	65.9
UK screening level ^c^	32–40 ^d^	-	-	-	-	82–330 ^d^	-

^a^ UDL: under detection limit; ^b^ Ross, S.M.; Wood M.D.; Copplestone, D.; Warriner, M. and Crook, P. [15] (determination by ICP-MS afteraqua regia extraction); ^c^ DEFRA, 2014 [16]; ^d^ Depending on the exposure parameters.

**Table 3:** Concentration (mg element/kg dry weight) of trace elements in the roots of the investigated plant species.

Location	Plant Species	Common Name	As	Cr	Cu	Mn	Ni	Pb	Zn
FG1	*Agrostis stolonifera*	Creeping bent	5	12	422	478	191	182	333
FG2	*Artemisia vulgaris*	Mugwort	2	2	82	88	105	10	190
FG3	*Argrostis capillaries*	Common bent	3	2	88	108	57	39	373
FG4	*Chamerion angustifolium*	Rosebay willowherb	1	2	19	25	6	3	53
FG5	*Agrostis tenuis*	Slender rush	3	10	731	456	317	256	558
FG6	*Dactylis glomerata*	Cocksfoot	2	10	77	212	28	27	180
FG7	*Urtica dioica*	Stinging nettle	2	7	61	59	16	15	124
FP1	*Galium aparine*	Cleavers	2	UDL	25	37	1	8	56
FP2	*Phalaris arundinacea*	Reed canary grass	3	2	14	34	3	14	111
FP3	*Heracleum sphondylium*	Hogweed	2	UDL	9	25	22	19	55
FP4	*Juncus inflexus*	Hard rush	5	8	43	192	8	19	88
FP5	*Impatiens glandulifera*	Himalayan balsam	4	UDL	38	42	15	12	119
MD1	*Plantago lanceolata*	Ribowort plantain	1	2	24	51	3	4	120
MD2	*Lolium multiflorum*	Italian Rye grass	4	1	22	207	3	13	215
MD3	*Juncus effusus*	Soft rush	24	4	27	89	9	31	116
MD4	*Chamerion angustifolium*	Rosebay willowherb	5	2	13	36	5	16	50
MD5	*Holcus lanatus*	Yorkshire fog	8	1	40	111	10	7	173
RS1	*Urtica dioica*	Stinging nettle	52	4	72	177	12	41	417
RS2	*Dactylis glomerata*	Cocksfoot	57	46	42	234	12	68	82
RS3	*Festuca pratensis*	Meadow fescue	32	3	23	225	5	48	83
RS4	*Glyceria maxima*	Reed sweetgrass	28	4	104	271	6	36	280
RS5	*Phleum pratense*	Timothy grass	29	3	47	584	16	26	161
RS6	*Lolium pratense*	Ryegrass	97	35	51	313	17	101	148
WH1	*Equisetum arvense*	Field horsetail	40	2	53	56	5	83	82
WH2	*Cynosurus cristatus*	Crested dog’s tail	4	7	32	582	9	13	118
WH3	*Filipendula ulmaria*	Meadowsweet	4	4	54	187	7	19	141
WH4	*Bistorta officinalis*	Bistort	103	3	28	485	12	50	52
Median			5	3	42	182	11	23	122
Min			1	UDL	9	25	1	3	50
Max			103	46	731	584	317	256	558

UDL: under detection limit.

**Table 4:** Concentration (mg element/kg dry weight) of trace elements in the shoots of the investigated plant species.

Location	Plant Species	Common Name	As	Cr	Cu	Mn	Ni	Pb	Zn
FG1	*Agrostis stolonifera*	Creeping bent	1.0	0.6	10.3	37.5	23.9	3.4	53.8
FG2	*Artemisia vulgaris*	Mugwort	1.7	0.3	12.3	81.2	24.1	2.0	189
FG3	*Argrostis capillaries*	Common bent	0.3	0.2	10.5	33.3	13.5	2.3	67.9
FG4	*Chamerion angustifolium*	Rosebay willowherb	0.7	UDL	8.5	21.6	2.6	0.2	39.8
FG5	*Agrostis tenuis*	Slender rush	1.3	0.7	58.7	83.3	52.8	27.8	147
FG6	*Dactylis glomerata*	Cocksfoot	0.0	0.2	9.3	98.5	5.3	2.7	39.5
FG7	*Urtica dioica*	Stinging nettle	0.3	0.3	18.2	9.1	1.4	1.3	17.7
FP1	*Galium aparine*	Cleavers	0.4	0.1	4.4	14.8	0.8	1.1	25.7
FP2	*Phalaris arundinacea*	Reed canary grass	0.8	0.2	11.6	201	5.4	3.4	84.6
FP3	*Heracleum sphondylium*	Hogweed	0.3	0.4	6.7	17.1	1.6	2.3	56.8
FP4	*Juncus inflexus*	Hard rush	3.4	0.4	12.1	28.0	0.9	1.6	28.1
FP5	*Impatiens glandulifera*	Himalayan balsam	0.3	0.2	7.7	55.7	2.8	1.8	41.8
MD1	*Plantago lanceolata*	Ribowort plantain	0.4	0.3	4.8	10.6	1.1	1.2	19.7
MD2	*Lolium multiflorum*	Italian Rye grass	0.6	0.2	6.3	105	1.4	3.3	44.0
MD3	*Juncus effusus*	Soft rush	1.1	0.4	5.7	9.6	1.0	0.3	39.6
MD4	*Chamerion angustifolium*	Rosebay willowherb	0.7	0.4	7.2	27.9	1.0	1.4	32.4
MD5	*Holcus lanatus*	Yorkshire fog	0.6	0.7	4.2	12.4	1.0	1.2	14.5
RS1	*Urtica dioica*	Stinging nettle	0.3	0.2	10.4	28.9	1.4	2.0	22.3
RS2	*Dactylis glomerata*	Cocksfoot	1.5	0.3	5.7	33.3	1.9	2.5	21.4
RS3	*Festuca pratensis*	Meadow fescue	1.3	0.5	8.6	123	2.0	4.1	37.0
RS4	*Glyceria maxima*	Reed sweetgrass	2.6	0.4	7.1	92.1	1.1	1.3	25.5
RS5	*Phleum pratense*	Timothy grass	6.2	0.8	8.8	278	4.3	6.6	40.1
RS6	*Lolium pratense*	Ryegrass	0.2	0.3	4.9	124	2.6	1.7	26.3
WH1	*Equisetum arvense*	Field horsetail	0.7	0.6	10.7	43.7	1.3	3.6	50.9
WH2	*Cynosurus cristatus*	Crested dog’s tail	0.4	0.6	5.2	178	3.7	1.6	35.3
WH3	*Filipendula ulmaria*	Meadowsweet	UDL	0.4	3.5	12.1	3.2	0.6	18.2
WH4	*Bistorta officinalis*	Bistort	1.0	0.8	8.8	540	3.5	0.6	63.4
Median			0.7	0.4	8.5	37.5	2.0	1.8	39.5
Min			UDL	UDL	3.5	9.1	0.8	0.2	14.5
Max			6.2	0.8	58.7	540	52.8	27.8	189

UDL: under detection limit.

**Table 5:** Bioaccumulation Factor of trace elements for the investigated plant species.

Location	Plant Species	Common Name	As	Cr	Cu	Mn	Ni	Pb	Zn
FG1	*Agrostis stolonifera*	Creeping bent	0.07	0.08	0.15	0.12	0.24	0.12	0.26
FG2	*Artemisia vulgaris*	Mugwort	0.02	0.01	0.02	0.03	0.12	0.01	0.19
FG3	*Argrostis capillaries*	Common bent	0.00	0.01	1.48	0.30	0.00	0.06	4.18
FG4	*Chamerion angustifolium*	Rosebay willowherb	0.11	0.05	0.10	0.06	0.11	0.02	0.29
FG5	*Agrostis tenuis*	Slender rush	0.09	0.08	0.59	0.13	0.30	0.29	0.30
FG6	*Dactylis glomerata*	Cocksfoot	0.05	0.10	0.19	0.28	0.19	0.08	0.83
FG7	*Urtica dioica*	Stinging nettle	0.04	0.05	0.10	0.06	0.12	0.04	0.22
FP1	*Galium aparine*	Cleavers	0.01	0.00	0.32	0.07	0.02	0.02	0.39
FP2	*Phalaris arundinacea*	Reed canary grass	0.02	0.02	0.08	0.05	0.04	0.03	0.25
FP3	*Heracleum sphondylium*	Hogweed	0.00	0.00	0.07	0.05	1.31	0.02	0.23
FP4	*Juncus inflexus*	Hard rush	0.10	0.07	0.67	0.45	0.15	0.14	0.69
FP5	*Impatiens glandulifera*	Himalayan balsam	0.08	0.00	0.64	0.10	0.27	0.09	1.00
MD1	*Plantago lanceolata*	Ribowort plantain	0.04	0.02	0.75	0.09	0.23	0.09	1.67
MD2	*Lolium multiflorum*	Italian Rye grass	0.00	0.01	0.58	4.22	0.00	0.02	7.69
MD3	*Juncus effusus*	Soft rush	0.09	0.03	0.77	0.26	1.51	0.16	1.63
MD4	*Chamerion angustifolium*	Rosebay willowherb	0.01	0.01	0.16	0.12	0.00	0.02	0.56
MD5	*Holcus lanatus*	Yorkshire fog	0.04	0.01	0.71	0.26	0.89	0.03	1.43
RS1	*Urtica dioica*	Stinging nettle	0.15	0.02	0.93	0.50	0.51	0.12	2.39
RS2	*Dactylis glomerata*	Cocksfoot	0.13	0.27	0.81	0.65	0.54	0.21	0.86
RS3	*Festuca pratensis*	Meadow fescue	0.36	0.02	0.01	0.12	0.01	0.04	0.06
RS4	*Glyceria maxima*	Reed sweetgrass	0.09	0.03	1.39	0.52	0.69	0.11	2.36
RS5	*Phleum pratense*	Timothy grass	0.03	0.01	0.53	0.26	0.00	0.04	1.76
RS6	*Lolium pratense*	Ryegrass	0.14	0.16	0.58	1.23	0.00	0.18	1.36
WH1	*Equisetum arvense*	Field horsetail	0.04	0.01	0.86	0.15	0.00	0.06	0.92
WH2	*Cynosurus cristatus*	Crested dog’s tail	0.04	0.06	0.24	1.86	0.96	0.09	1.23
WH3	*Filipendula ulmaria*	Meadowsweet	0.01	0.03	1.07	0.40	0.00	0.08	1.32
WH4	*Bistorta officinalis*	Bistort	0.82	0.03	0.52	1.23	0.72	0.34	0.52
Median			0.04	0.02	0.58	0.26	0.15	0.08	0.86
Min			0.00	0.00	0.01	0.03	0.00	0.01	0.06
Max			0.82	0.27	1.48	4.22	1.51	0.34	7.69

**Table 6:** Translocation factor of trace elements for the investigated plant species.

Location	Plant Species	Common Name	As	Cr	Cu	Mn	Ni	Pb	Zn
FG1	*Agrostis stolonifera*	Creeping bent	0.19	0.01	0.02	0.08	0.13	0.02	0.16
FG2	*Artemisia vulgaris*	Mugwort	0.92	0.15	0.15	0.92	0.22	0.20	0.99
FG3	*Argrostis capillaries*	Common bent	0.08	0.09	0.12	0.31	0.23	0.06	0.18
FG4	*Chamerion angustifolium*	Rosebay willowherb	0.48	0.02	0.44	0.85	0.41	0.05	0.74
FG5	*Agrostis tenuis*	Slender rush	0.39	0.07	0.08	0.18	0.16	0.11	0.26
FG6	*Dactylis glomerata*	Cocksfoot	0.00	0.02	0.12	0.46	0.19	0.10	0.22
FG7	*Urtica dioica*	Stinging nettle	0.12	0.05	0.30	0.16	0.08	0.09	0.14
FP1	*Galium aparine*	Cleavers	0.16	0.00	0.17	0.39	0.59	0.13	0.45
FP2	*Phalaris arundinacea*	Reed canary grass	0.27	0.10	0.83	5.42	1.76	0.25	0.76
FP3	*Heracleum sphondylium*	Hogweed	0.16	1.94	0.75	0.69	0.07	0.12	1.03
FP4	*Juncus inflexus*	Hard rush	0.63	0.05	0.28	0.15	0.12	0.08	0.32
FP5	*Impatiens glandulifera*	Himalayan balsam	0.07	0.54	0.20	1.31	0.19	0.15	0.34
MD1	*Plantago lanceolata*	Ribowort plantain	0.28	0.15	0.20	0.21	0.43	0.29	0.16
MD2	*Lolium multiflorum*	Italian Rye grass	0.56	0.13	0.28	0.51	0.50	0.25	0.20
MD3	*Juncus effusus*	Soft rush	0.04	0.09	0.21	0.11	0.11	0.01	0.34
MD4	*Chamerion angustifolium*	Rosebay willowherb	0.14	0.17	0.58	0.78	0.18	0.09	0.65
MD5	*Holcus lanatus*	Yorkshire fog	0.07	0.94	0.10	0.11	0.10	0.16	0.08
RS1	*Urtica dioica*	Stinging nettle	0.01	0.06	0.15	0.16	0.12	0.05	0.05
RS2	*Dactylis glomerata*	Cocksfoot	0.03	0.01	0.13	0.14	0.16	0.04	0.26
RS3	*Festuca pratensis*	Meadow fescue	0.04	0.12	0.38	0.55	0.44	0.09	0.44
RS4	*Glyceria maxima*	Reed sweetgrass	0.10	0.09	0.07	0.34	0.18	0.04	0.09
RS5	*Phleum pratense*	Timothy grass	0.21	0.28	0.19	0.48	0.27	0.25	0.25
RS6	*Lolium pratense*	Ryegrass	0.00	0.01	0.10	0.40	0.15	0.02	0.18
WH1	*Equisetum arvense*	Field horsetail	0.02	0.26	0.20	0.78	0.26	0.04	0.06
WH2	*Cynosurus cristatus*	Crested dog’s tail	0.09	0.08	0.16	0.31	0.40	0.12	0.30
WH3	*Filipendula ulmaria*	Meadowsweet	0.01	0.11	0.06	0.06	0.44	0.03	0.13
WH4	*Bistorta officinalis*	Bistort	0.01	0.25	0.31	1.11	0.29	0.01	1.22
Median			0.10	0.09	0.19	0.39	0.19	0.09	0.26
Min			0.00	0.00	0.02	0.06	0.07	0.01	0.05
Max			0.92	1.94	0.83	5.42	1.76	0.29	1.22

**Table 7:** A comparison of shoot-borne trace elements (mg element/kg dry weight) between this study and other reports.

Element	Misra and Mani [36]	MacLean et al. [37]	This Study
As	0.02–7	-	0–6.18
Cr	0.2–1	1.22	0.04–0.78
Cu	4.15	12.3	3.46–58.7
Mn	15–100	-	9.14–540
Ni	1	4.08	0.75–52.8
Pb	1–13	1.54	0.16–27.8
Zn	8–100	28.4	14.5–189
